# Pharmacodynamics and Pharmacokinetics of Injectable Pimobendan and Its Metabolite, O-Desmethyl-Pimobendan, in Healthy Dogs

**DOI:** 10.3389/fvets.2021.656902

**Published:** 2021-08-20

**Authors:** Poonavit Pichayapaiboon, Lalida Tantisuwat, Pakit Boonpala, Nakkawee Saengklub, Tussapon Boonyarattanasoonthorn, Phisit Khemawoot, Anusak Kijtawornrat

**Affiliations:** ^1^Department of Physiology, Faculty of Veterinary Science, Chulalongkorn University, Bangkok, Thailand; ^2^Department of Physiology, Faculty of Pharmacy, Mahidol University, Bangkok, Thailand; ^3^Faculty of Medicine Ramathibodi Hospital, Chakri Naruebodindra Medical Institute, Mahidol University, Bangkok, Thailand

**Keywords:** cardiovascular, dog, intravenous, ODMP, pharmacodynamics, pharmacokinetics, pimobendan

## Abstract

**Objectives:** This study was designed to thoroughly evaluate the effects of bolus pimobendan at a dose of 0.15 mg/kg on cardiac functions, hemodynamics, and electrocardiographic parameters together with the pharmacokinetic profile of pimobendan and its active metabolite, o-desmethyl-pimobendan (ODMP), in anesthetized dogs.

**Methods:** Nine beagle dogs were anesthetized and instrumented to obtain left ventricular pressures, aortic pressures, cardiac outputs, right atrial pressures, pulmonary arterial pressures, pulmonary capillary wedge pressures, electrocardiograms. After baseline data were collected, dogs were given a single bolus of pimobendan, and the pharmacodynamic parameters were obtained at 10, 20, 30, 60, and 120 min. Meanwhile, the venous blood was collected at baseline and 2, 5, 10, 20, 30, 60, 120, 180, 360, and 1,440 min after administration for the determination of pharmacokinetic parameters.

**Results:** Compared with baseline measurements, the left ventricular inotropic indices significantly increased in response to intravenous pimobendan, as inferred from the maximum rate of rise in the left ventricular pressure and the contractility index. Conversely, the left ventricular lusitropic parameters significantly decreased, as inferred from the maximum rate of fall in the left ventricular pressure and the left ventricular relaxation time constant. Significant increases were also noted in cardiac output and systolic blood pressure. Decreases were observed in the systemic vascular resistance, pulmonary vascular resistance, left ventricular end-diastolic pressure, pulmonary capillary wedge pressure, right atrial pressure, and pulmonary arterial pressure. The heart rate increased, but the PQ interval decreased. There was no arrhythmia during the observed period (2 h). The mean maximum plasma concentration (in μg/L) for ODMP was 30.0 ± 8.8. Pimobendan exerted large volume of distribution ~9 L/kg.

**Conclusions:** Intravenous pimobendan at the recommended dose for dogs increased cardiac contraction and cardiac output, accelerated cardiac relaxation but decreased both vascular resistances. These mechanisms support the use of injectable pimobendan in acute heart failure.

## Introduction

Pimobendan, a benzimidazole-pyridazinone derivative, is widely used for the management of both asymptomatic and symptomatic canine congestive heart failure [CHF; (1, 2)]. It acts as an inhibitor of phosphodiesterase III (PDE-3) and as a calcium sensitizer, and it has two major effects on the cardiovascular system (3). First, pimobendan increases the intracellular cAMP content in both myocytes and vascular smooth muscle cells, resulting in increased cardiac contraction and promotion of vascular relaxation, respectively. Increased cardiac contraction has been demonstrated previously in the excised, cross-circulated dog heart without an excessive increase in myocardial oxygen consumption (4). Second, pimobendan increases the affinity of troponin C to intracellular calcium, causing a positive inotropic effect (5).

Several clinical trials have supported the use of pimobendan in veterinary medicine, especially in dogs with myxomatous mitral valve degeneration (MMVD) stages C and D (6, 7) and CHF caused by dilated cardiomyopathy (8). The previous guidelines for the diagnosis and treatment of canine chronic valvular heart disease have recommended the use of pimobendan in addition to angiotensin-converting enzyme inhibitors and diuretics for CHF treatment (9). Recently, clinical trials have supported the use of pimobendan in asymptomatic MMVD (10, 11). These newer clinical trials led to updated guidelines for the diagnosis and treatment of MMVD in dogs, published by the American College of Veterinary Internal Medicine; the update included the use of pimobendan in dogs with MMVD stage B2 (2).

Previously, pimobendan had been supplied in the form of a capsule or chewable tablet. In dogs, pimobendan may take 2–4 h to reach the maximum effect when given orally (12), which is not ideal for emergency cases of acute CHF. Currently, injectable pimobendan is available in several countries (e.g., United Kingdom, Australia). However, limited data are available in dogs. A single bolus of pimobendan was recently investigated in anesthetized healthy dogs for 1 h; the treatment increased the maximum rate of rise (dP/dt_max_) in the left ventricular pressure (LVP) but decreased the left ventricular end-diastolic pressure (LVEDP) (13). Surprisingly, there was no effect on the maximum rate of fall (dP/dt_min_) of the LVP and heart rate (HR). Though most studies have focused on the cardiac function of dogs in response to intravenous pimobendan, no data are available about the effects of injectable pimobendan on vascular resistances, pulmonary capillary wedge pressure (PCWP), and pharmacokinetics (PK). Therefore, this study was conducted to more thoroughly evaluate the effects of a single bolus of pimobendan at a dose of 0.15 mg/kg on cardiac functions, hemodynamics, and electrocardiographic parameters together with the PK profile of pimobendan and its active metabolite, o-desmethyl-pimobendan (ODMP), in anesthetized dogs.

## Materials and Methods

### Animals

This study was approved by the Institutional Animal Care and Use Committee of Chulalongkorn University Laboratory Animal Center (protocol number 1873019). All experimental animal procedures were performed in compliance with ethical principles and guidelines for the use of animal for scientific purposes, the Animals for Scientific Purposes Act 2015, and the Guide for the Care and Use of Laboratory Animals (14). Nine healthy, mature beagles (*Canis familiaris*) of either gender were housed in a dog run maintained at a temperature of 22 ± 1°C, a relative humidity of 50 ± 20%, and a 12:12 h light:dark cycle. Physical examination, lead II electrocardiogram (ECG), complete blood count, and blood chemistry profiles (i.e., blood urea nitrogen, serum creatinine, serum alkaline phosphatase, serum alanine aminotransferase, and serum aspartate aminotransferase measures) were performed to evaluate healthy status in all dogs before beginning the experiment. Dogs were excluded from the study if there was evidence of clinically important systemic or cardiovascular disease upon initial assessment.

### Surgical Procedure and Instrumentation

On the experiment day, dogs were given carprofen (4 mg/kg subcutaneously) and cefazolin (25 mg/kg intravenously). Then, an intravenous bolus of propofol (6–8 mg/kg) was given and was followed by endotracheal tube intubation. The animals were ventilated mechanically with 100% O_2_ via a volume-cycled ventilator (Veterinary Anesthesia Ventilators Model 2000^TM^, Hallowell EMC^©^, Pittsfield, MA, USA) at a rate of 8–12 breaths/min and a tidal volume of ~20 mL/kg, sustaining the arterial partial pressure of carbon dioxide at 35–45 mmHg and that of O_2_ at >85 mmHg. Body temperature was maintained at 36.5–37°C by a heat therapy pump (T/Pump® Model TP-500, Gaymar^©^, Orchard Park, NY, USA). A surgical plane of anesthesia was maintained by inhalation of isoflurane, and the end-tidal inhalant concentration was maintained at 1.4–1.6%.

Each animal was shaved and scrubbed at the surgical areas and prepared by aseptic technique (at the left jugular area). All catheterization procedures were performed under fluoroscopic guidance, as previously described (15). A 5 French Mikro-Tip catheter pressure transducer (Millar, Inc., Houston, TX, USA) was inserted into the left carotid artery and advanced to the left ventricle for measuring the LVP. A 5 French thermodilution catheter (Edwards Lifesciences, Irvine, CA, USA) was inserted into the left jugular vein and advanced into the pulmonary artery to permit simultaneous continuous monitoring of the right atrial pressure (RAP) and pulmonary arterial pressure (PAP) as well as intermittent determination of PCWP and cardiac output (CO) using a thermodilution technique. A lead II ECG was obtained. After stabilization, at ~30 min, baseline data were recorded. Then, a single bolus of pimobendan (Vetmedin injectable solution for dogs, 0.75 mg/mL, Boehringer Ingelheim, Ingelheim am Rhein, Germany) at the recommended dose from the manufacturer (0.15 mg/kg) was intravenously injected, and injection was followed by an observation period of 2 h. The period of 2 h was chosen because the plasma elimination half-life of ODMP obtained from the package insert was 2.0 ± 0.3 h. The ECGs and pressures were recorded throughout the experiment with an EMKA-IOX system (IOX 2.10.8.6, EMKA Technologies, Paris, France) and were stored on a hard drive for later analysis. All parameters were analyzed at baseline and 10, 20, 30, 60, and 120 min after the beginning of the injection. The CO was measured at baseline and at 10, 20, 30, 60, and 120 min by a standard bolus thermodilution technique using 25°C normal saline. At the end of experiment (i.e., 2 h after pimobendan administration), all catheters were removed and the vessels were sutured with 6-0 monofilament non-absorbable polypropylene suture materials. Tissues and muscles were sutured with absorbable 3-0 suture materials. Skin was closed with monofilament polyamide suture. Carprofen (4 mg/kg once a day) and cefazolin (25 mg/kg twice daily) were administered orally for 3 and 7 days, respectively.

### PK of Intravenous Pimobendan

Simultanesouly with the pharmacodynamic study in the previous section, three milliliters of blood was collected via the cephalic vein at baseline and 2, 5, 10, 20, 30, 60, 120, 180, 360, and 1,440 min after administration of a single bolus of pimobendan. The blood samples were collected in lithium heparin-coated blood tubes; they were centrifuged at 5,000 × *g* and 4°C for 10 min to separate plasma within 1 h after collection. The plasma samples were stored at −20°C for additional analysis. At the time of analysis, plasma samples were thawed at room temperature; then, 50 μL of each sample was mixed with 200 μL of absolute methanol containing the internal standard (glycyrrhizin 100 ng/mL). The mixtures were then vortex mixed and centrifuged at 10,000 × *g* for 10 min. After centrifugation, 10 μL of supernatant was collected and injected into the liquid chromatography tandem mass spectrometry system.

Liquid chromatography tandem mass spectrometry analysis was conducted with modifications from previously described by Bell et al. (3) and Yata et al. (12). In this study, the Nexera ultra high-performance liquid chromatography and 8060 triple quadrupole mass spectrometers (Shimadzu Co., Ltd., Kyoto, Japan) were used for the liquid chromatography tandem mass spectrometry module, and the Synergi Fusion-RP C18 column (Phenomenex, Inc., Torrance, CA, USA) was used for the stationary phase. The oven temperature was maintained at 40°C during analysis.

A mobile phase consisted of 0.2% formic acid in water and absolute methanol. The gradient started with 10% methanol at 0–0.5 min; then, the concentration of methanol was increased to 90% during 0.5–1.5 min and was maintained at 90% until 3.0 min after injection. The gradient was reduced to 10% at 3.0–4.0 min and was maintained at 10% until 5.0 min. The retention times of pimobendan, ODMP, and the internal standard were 2.12, 1.58, and 2.05 min, respectively, and the mass-to-charge ratios of each compound were 335/319, 321.10/305.05, and 821.25/350.90 *m/z*, respectively. The lower limit for detection was 0.09 μg/L for both pimobendan and ODMP. The standard curves for pimobendan and ODMP indicated a good linearity range of 0.09–100 and 0.09–200 μg/L, respectively (*R*^2^ > 0.99). The intra-day and inter-day precision and accuracy were determined at concentrations from 1 to 100 μg/L for pimobendan and from 1 to 200 μg/L for ODMP. The precision (% CV) ranged from 4.04 to 8.96% for pimobendan and from 4.78 to 9.43% for ODMP. The accuracy ranged from 92.70 to 100.52% and 93.10 to 109.40% for pimobendan and ODMP, respectively. Percent recoveries of the both compounds were more than 70%.

### Data Analysis

All recorded data were analyzed by EMKA_ECG Auto software (ECG Auto 3.5.5.12, EMKA Technologies, Paris, France). The systemic vascular resistance (SVR) and the pulmonary vascular resistance (PVR) were calculated as previously described (15). The contractility Index, or CI, was defined as the ratio of maximal rate of rise in the LVP over the LVP at that point and was calculated from the following equation: CI = (dP/dt_max_) ÷ LVP. The tau, or the exponential decline of ventricular pressure during isovolumic relaxation, was calculated with the method by Raff and Glantz (16). The CO was calculated from integration of the area under curve by the CO machine (Baxter COM-2 cardiac output computer, Baxter Healthcare^©^, Round Lake, IL, USA). Electrocardiographic data were analyzed for rhythm—including PQ interval, QRS complex, and QT interval—and rate. The value of each parameter was averaged from cardiac cycles over 60 s of each time point. The corrected QT interval was calculated using Van der Water's correction formula (17). The PK analysis was conducted by non-compartmental model using PK solution software (Summit Research Services, CO, USA). C_max_ and T_max_ were directly observed from plasma concentration-time curves of each dog. AUC_0−t_ was calculated by using trapezoidal rule and extrapolated to time infinity by the equation AUC_0−inf_ = AUC_0−t_ + (C_t_/k_el_), where C_t_ is the last observed plasma concentration after dosing and k_el_ is the elimination rate constant, calculated using the log-linear slope of the terminal phase of the concentration–time curve. Mean residence time (MRT) was calculated as AUMC_0−inf_ /AUC_0−inf_, where AUMC_0−inf_ is area under the first moment concentration-time curve. Volume of distribution (Vd) was equal to CL/k_el_ and total clearance (CL) was calculated as dose/ AUC_0−inf_. The terminal elimination half-life was determined by dividing 0.693 by k_el_.

### Statistical Analysis

In this study, the power analysis was performed to calculate sample size using G-power program and the information used in the program was based on previous publication (18). Pharmacodynamic data are presented as mean ± standard error of the mean (SEM) while pharmacokinetic parameters were presented as mean ± standard deviation (SD). Statistical analyses were performed with commercially available software. Normal distribution of continuous data was assessed by the Shapiro-Wilk test. Differences among time points were determined using one-way analysis of variance with repeated measures followed by Dunnett's *post hoc* analysis. Values of *P* < 0.05 were considered significant for all analyses.

## Results

In this study, the physical examination, complete blood count, and blood chemistry profiles of all dogs before the beginning of the study were within normal limits. During the experiment, all parameters at each time point were measurable from all dogs.

### Acute Effects of a Single Bolus of Pimobendan on Left Ventricular Functions

**Figure 1:**
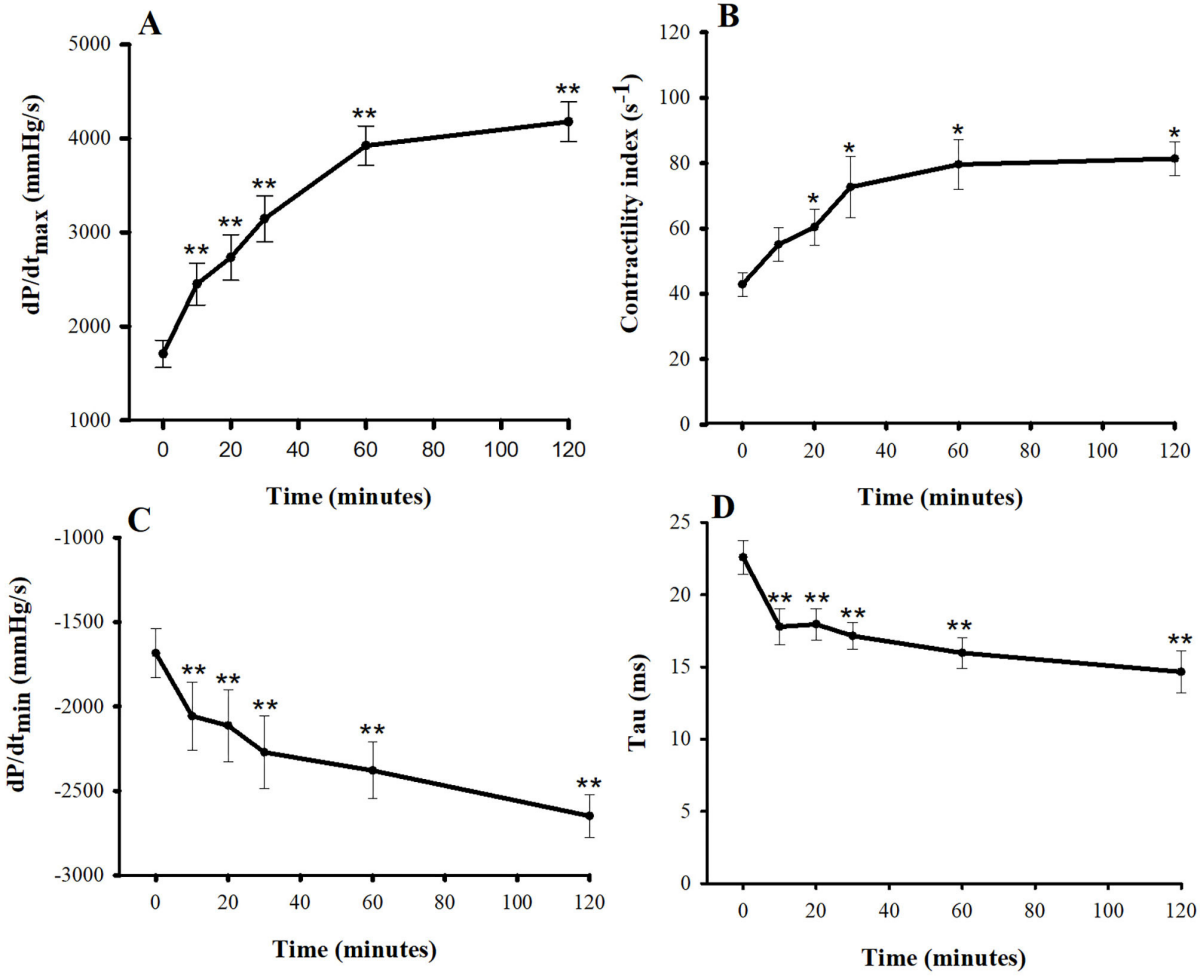
Plots of inotropic effects—**(A)** the maximum rate of rise in the left ventricular pressure (dP/dt_max_) and **(B)** contractility index—and of lusitropic effects—**(C)** the maximum rate of decrease in the left ventricular pressure (dP/dt_min_) and **(D)** tau vs. time (min) after a single bolus of intravenous pimobendan (0.15 mg/kg) in healthy, anesthetized beagle dogs. Values are presented as mean ± standard error of mean. ^*^P < 0.05, ^**^P < 0.01.

Inotropic and lusitropic properties of the left ventricle were assessed by parameters obtained from the LVP curve (Figures 1A–D). The dP/dt_max_ and CI at baseline were 1,708 ± 144 mmHg/s and 43 ± 3.6 s^−1^, respectively. Compared with baseline, the dP/dt_max_ increased in response to a single bolus of pimobendan significantly at 10 min (43.4%, *P* < 0.01) while the CI was significantly increased at 20 min (39.5%, *P* < 0.05). Both dP/dt_max_ and CI continued to increase until 60 min after injection and remained quite stable from this point to the 120 min. The dP/dt_min_ decreased after injection (i.e., became more negative) and differed significantly from baseline at 10 min after the injection (-22.1%, *P* < 0.01). Conversely, the tau at baseline was 22.6 ± 1.1 ms, and it decreased significantly at 10 min after the injection (−21.2%, *P* < 0.05). These lusitropic indices continued to change until the end of experiment.

### Acute Effects of a Single Bolus of Pimobendan on Hemodynamics

**Figure 2:**
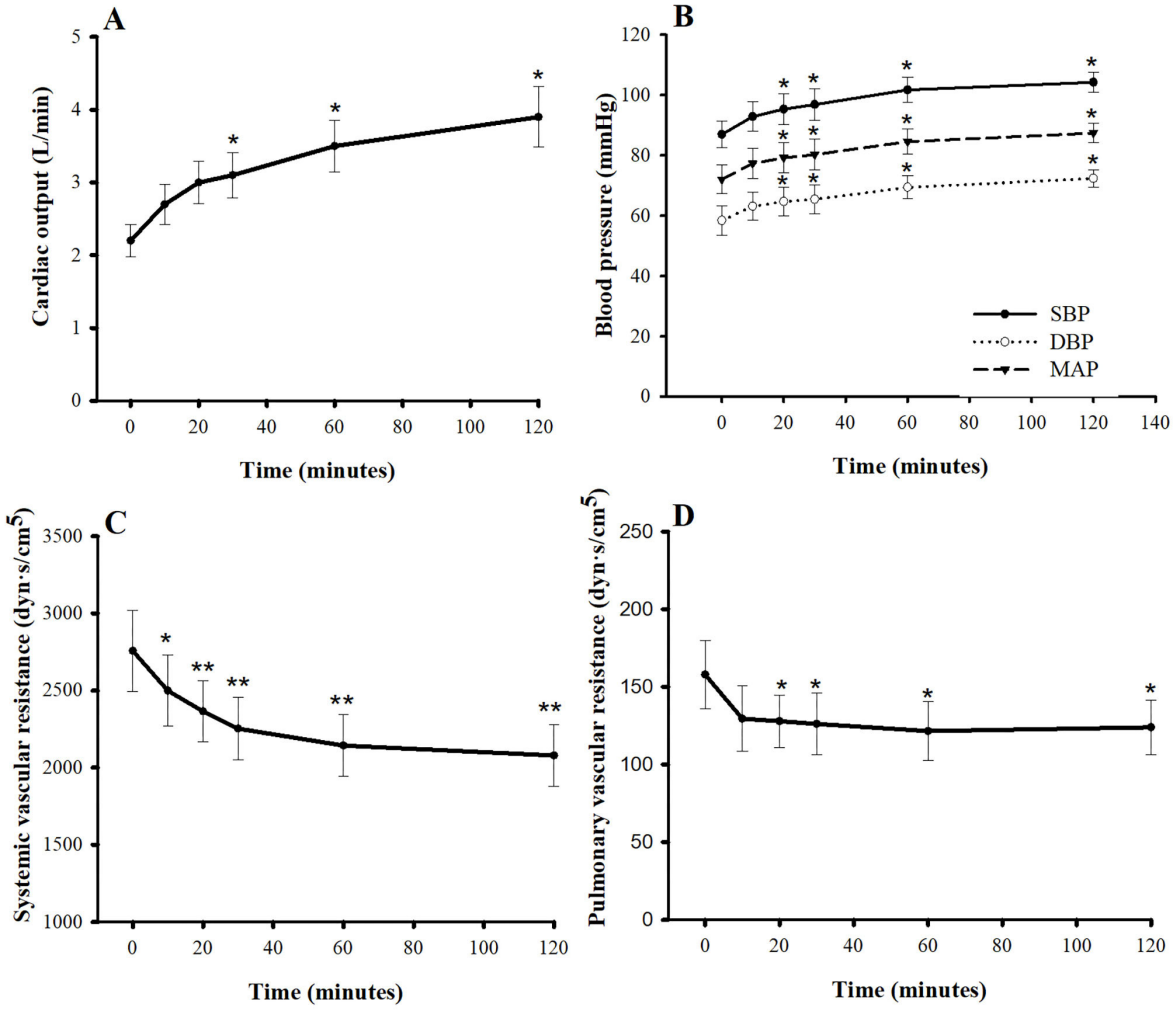
Plots of **(A)** cardiac output, **(B)** blood pressures, **(C)** systemic vascular resistance, and **(D)** pulmonary vascular resistance vs. time (min) after a single intravenous bolus of pimobendan (0.15 mg/kg) in healthy, anesthetized beagle dogs. Values are presented as mean ± standard error of mean. SBP, systolic blood pressure; DBP, diastolic blood pressure; MBP, mean blood pressure. ^*^P < 0.05, ^**^P < 0.01.

**Figure 3:**
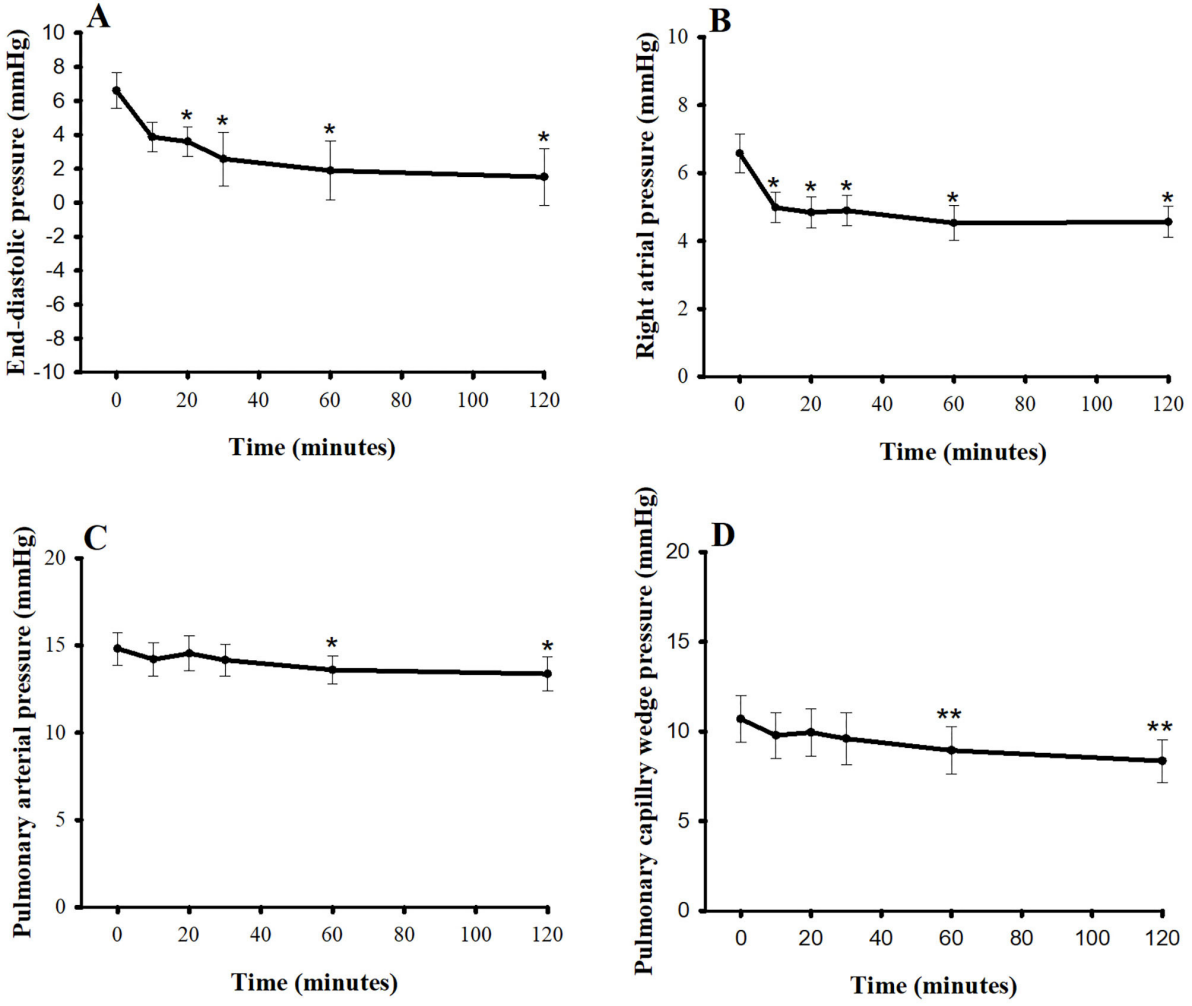
Plots of **(A)** left ventricular end-diastolic pressure, **(B)** right atrial pressure, **(C)** pulmonary arterial pressure, and **(D)** pulmonary capillary wedge pressure vs. time (min) after a single intravenous bolus of pimobendan (0.15 mg/kg) in healthy, anesthetized beagle dogs. Values are presented as mean ± standard error of mean. ^*^P < 0.05, ^**^P < 0.01.

Figures 2, 3 show the hemodynamic parameters of pimobendan both before and after injection. When dogs were given pimobendan, CO continuously increased from baseline, and the change became significantly different from baseline at 30 min (40.9%, *P* < 0.05). The CO continued to increase slowly until the end of experiment at 2 h (77.3%, *P* < 0.05). The systolic blood pressure (SBP), mean blood pressure (MBP), and the diastolic blood pressure (DBP) gradually increased, and the difference from baseline became significant at 20 min (9.5, 10.0, and 10.8%, respectively, compared with baseline; *P* < 0.05). All blood pressures continued to increase until 60 min after injection (16.9, 17.5, and 18.8% greater than baseline, respectively; *P* < 0.05), and they remained relatively stable until the end of experiment (19.8, 21.4, and 24.0% greater than baseline, respectively; *P* < 0.05). SVR was significantly lower compared with baseline at 10 min (−9.3%, *P* < 0.05) and continued to decrease until 60 min (−22.2%, *P* < 0.01). Thereafter, SVR slowly decreased until the end of experiment (−24.6% difference from baseline, *P* < 0.01). The PVR decreased significantly from baseline at 10 min after injection (−20.4%, *P* < 0.05) and remained stable until the end of experiment (−31.7%, *P* < 0.05). Notably, the LVEDP decrease differed significantly from baseline at 20 min after injection (−54.5%, *P* < 0.05), and the right atrial pressure was significantly lower than baseline at 10 min after injection (−24.2%, *P* < 0.05); both continued to decrease slowly until the end of experiment. Both PAP and PCWP decreased gradually and became significantly different from baseline at 60 min [−7.6% (*P* < 0.05) and −14.4% (*P* < 0.01), respectively] and 120 min [−10.0% (*P* < 0.05) and −22.4% (*P* < 0.01), respectively].

### Acute Effects of a Single Bolus of Pimobendan on ECGs

**Figure 4:**
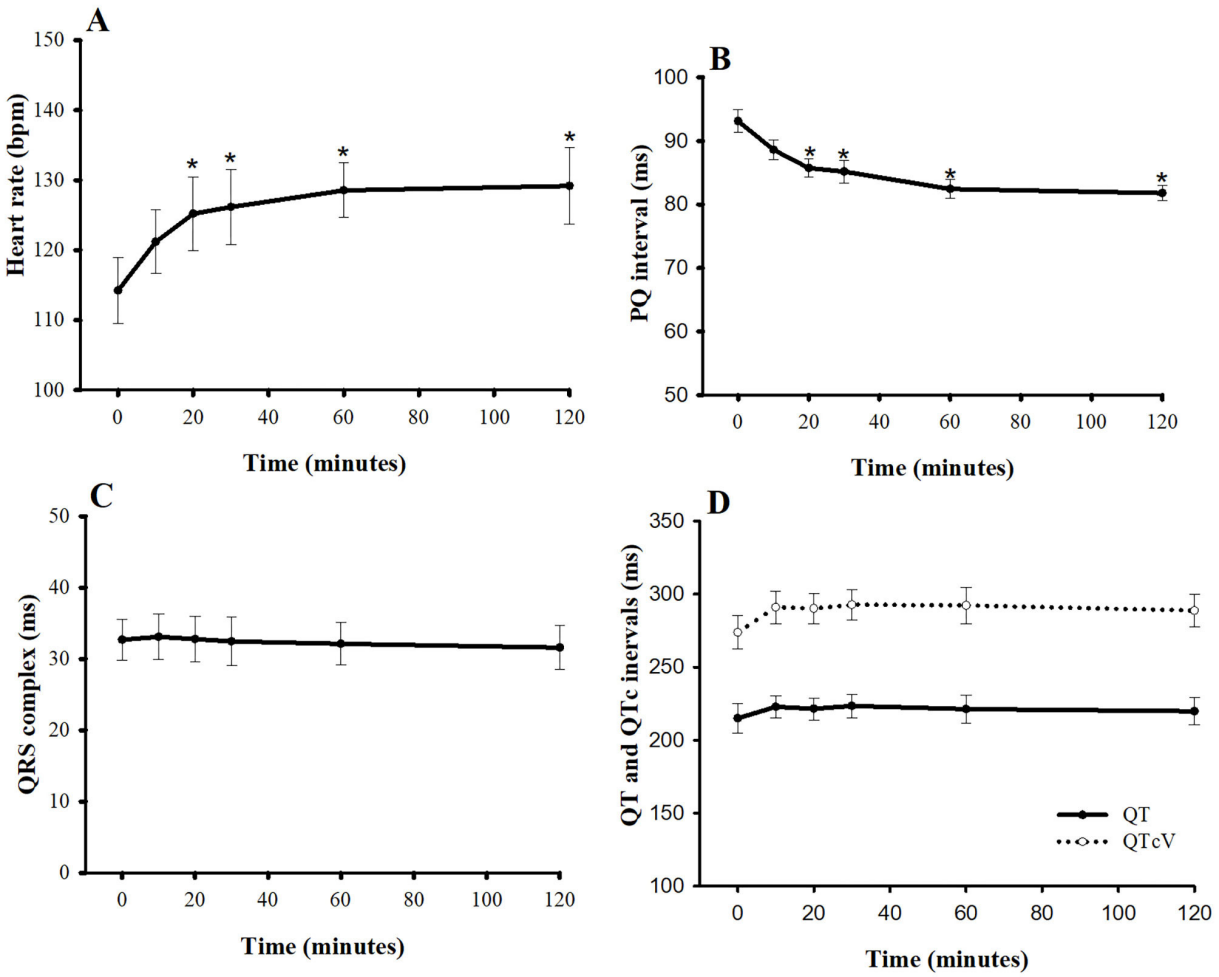
Plots of **(A)** heart rate, **(B)** PQ interval, **(C)** QRS complex, and **(D)** QT and corrected QT intervals vs. time (min) after a single intravenous bolus of pimobendan (0.15 mg/kg) in healthy, anesthetized beagle dogs. Values are presented as mean ± standard error of mean. QTcV is the QT interval corrected for heart rate by Van de Water's formula (18). ^*^P < 0.05.

At baseline, the average HR and average PQ interval were 114 ± 5 bpm and 93 ± 1.8 ms, respectively (Figure 4). Pimobendan significantly increased HR at 20 min after injection (9.6%; *P* < 0.05), and HR continued to increase slowly until it reached its peak at 60 min (13.2% difference from baseline, *P* < 0.05). After that, the HR was stable until the end of the experiment (12.3% difference from baseline, *P* < 0.05). The PQ interval gradually shortened and became significantly different from baseline at 20 min after injection (−7.5%, *P* < 0.05). The interval then slowly decreased to a maximum decrease at 60 min (−11.8%, *P* < 0.05). Thereafter, it was relatively stable until the end of experiment (−11.8%, *P* < 0.05) compared with the baseline value. The QRS complex, QT interval, and corrected QT interval were unaltered. Interestingly, no arrhythmia was observed after injection until 2 h post-injection.

### PK of Pimobendan and ODMP

**Figure 5:**
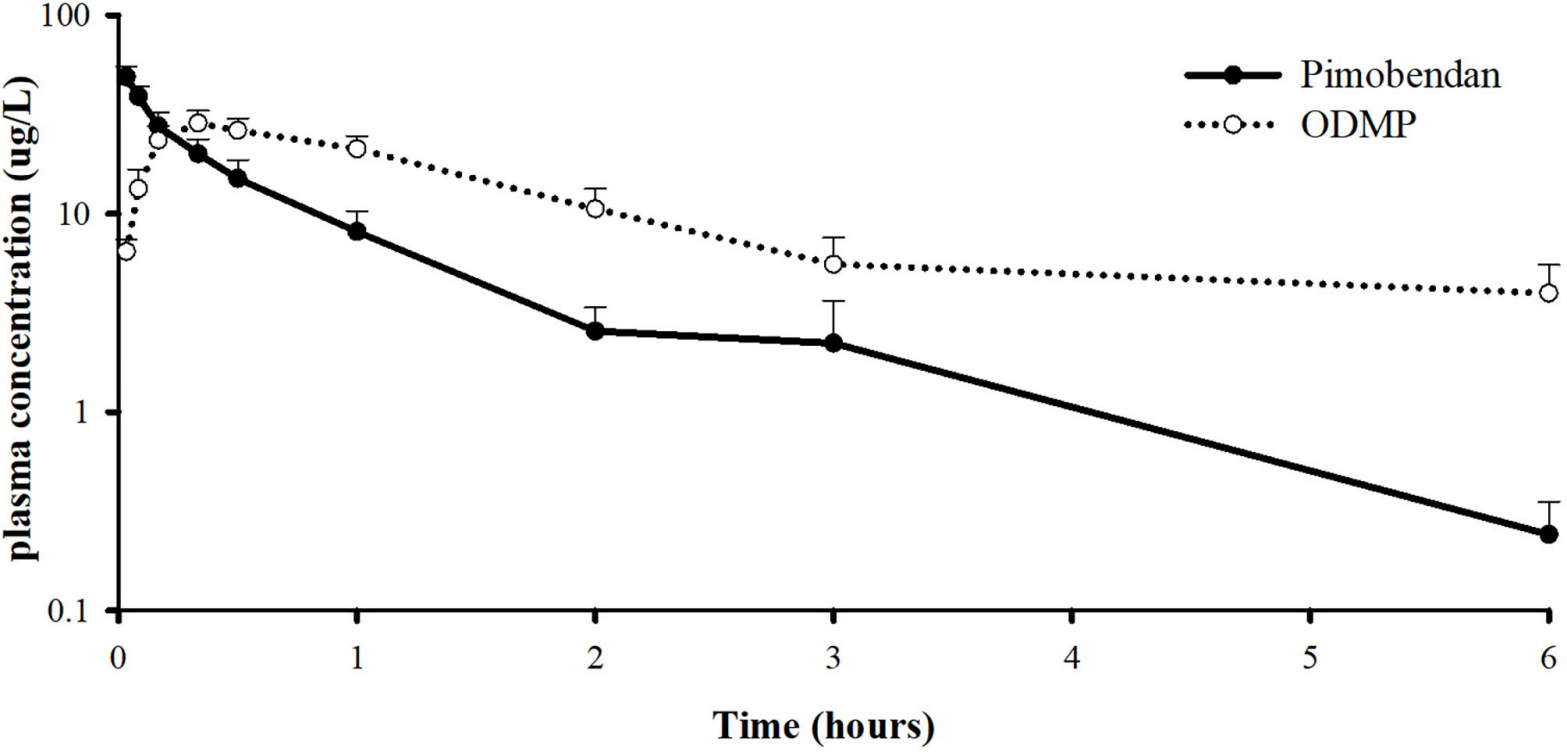
Log scale of plasma concentration-time profile of pimobendan and its active metabolite, o-desmethyl-pimobendan (ODMP) for healthy, anesthetized beagle dogs given a single bolus of pimobendan (0.15 mg/kg).

The plasma concentrations of pimobendan and its active metabolite, ODMP, after intravenous injection of pinobendan 0.15 mg/kg were plotted vs. time as the mean ± SD in semi-log scale (Figure 5). The mean plasma concentration of pimobendan sharply decreased from 48.86 ± 13.92 μg/L at 2 min to 8.12 ± 4.91 μg/L in the first hour after injection. Then, it gradually decreased through the end of the experiment. Conversely, the plasma concentration of ODMP gradually increased from 0 μg/L at baseline and reached its maximal plasma concentration of 30.0 ± 8.8 μg/L within 20 min after injection of the parent drug. After it reached its maximal plasma concentration, the active metabolite began to decline slowly until the end of the experiment. The PK properties of intravenous pimobendan and ODMP are presented in Table 1. Pimobendan had large Vd ~9 L/kg. The half-life of pimobendan is shorter than ODMP, and also MRT of pimobendan is faster than ODMP.

**Table 1:** Pharmacokinetic profiles of pimobendan and o-desmethyl-pimobendan (in mean ± SD) after a single bolus of intravenous pimobendan (0.15 mg/kg) in healthy dogs.

**Parameters**	**Pimobendan**	**O-desmethyl-pimobendan**
C_max_ (μg/L)	N/A	30.0 ± 8.8
T_max_ (h)	N/A	0.33 ± 0.1
AUC_0−t_ (μg.h/L)	30.9 ± 7.2	66.4 ± 17.5
AUC_0−inf_ (μg.h/L)	31.3 ± 7.4	88.4 ± 26.3
MRT (h)	2.2 ± 0.6	4.9 ± 2.3
Vd (L/kg)	8.9 ± 4.9	N/A
CL (L/kg/h)	5.8 ± 2.3	N/A
Half-life (h)	1.0 ± 0.4	2.8 ± 1.6

*AUC, area under the concentration-vs.-time curve; C_max_, maximum plasma concentration; CL, total clearance; MRT, mean residence time; N/A, not applicable; T_max_, time to maximum concentration; Vd, volume of distribution*.

## Discussion

To our knowledge, this was the first study conducted to determine the acute effects of a bolus of pimobendan at the manufacturer's recommended dose on both PK and pharmacodynamics (PD) (i.e., cardiovascular functions, hemodynamics, ECGs) in healthy dogs anesthetized with isoflurane.

Previous studies in anesthetized and conscious dogs have shown that various doses of intravenous pimobendan increase cardiac contractility, as evaluated by the left ventricular dP/dt_max_ (13, 18, 19) and the slope of the left ventricular end-systolic pressure-volume relationship (20). Similarly, the left ventricular contractility measured in this study increased significantly compared with the baseline, as assessed by the dP/dt_max_ and CI. The dP/dt_max_ is affected by loading conditions, HR, and myocardial contractility (21), but the CI is considered less load dependent (22, 23). In our study, the dP/dt_max_ achieved statistical significance at 10 min after injection, whereas the CI became significant later (at 30 min after injection). This result was supported by a previous telemetry study in beagles, which demonstrated that dP/dt_max_ is a robust, reliable, and sensitive index for assessing inotropic effects of a drug when the loading and the HR are constant (24). The increase in left ventricular contractility in our study could be explained by the inhibition of PDE-3 and the sensitization of troponin C to intracellular calcium (5).

In this study, the effect of pimobendan on cardiac relaxation was assessed by dP/dt_min_ and tau. These indices are obtained during isovolumetric relaxation, not after ventricle has filled completely (25). The left ventricular dP/dt_min_ is determined by lusitropy, reduction in HR, diastolic systemic arterial pressure, structural properties of the myocardium, and constriction of the pericardium or pericardial effusion (26). In response to intravenous pimobendan, both tau and the dP/dt_min_ decreased (became more negative). The observed change in tau (i.e., positive lusitropy) may result from an inhibitory effect of pimobendan on PDE-3, which accelerates phosphorylation of phospholamban; therefore, more calcium is resequestrated through the Ca^2+^-ATPase channel of the sarco/endoplasmic reticulum, causing faster relaxation (27). This result is also in accordance with previous studies, in which tau and the dP/dt_min_ decreased significantly when pimobendan was given to anesthetized dogs (4).

As expected, the increase in left ventricular contraction resulted in increased CO. The SBP in our study also increased. In general, the SBP is determined by stroke volume, stiffness of the aorta, and the DBP. The increase in SBP in our study may be due to an increase in stroke volume, a hypothesis supported by the study of van Meel and Diederen (19) that demonstrated an increase in the stroke volume by ~59% in response to pimobendan injection. Because blood pressure is a product of CO and total peripheral resistance, an elevation of CO must overcome the reduction of total peripheral resistance observed in our study. The SVR in our study was remarkably decreased at 10 min, whereas the PVR was slightly decreased at 20 min. The SVR is determined mainly by the diameter of the blood vessels. Pimobendan and its active metabolite possess PDE-3 inhibitory effects that may dilate the resistant vessels (i.e., small arteries and arterioles). Although the SVR decreased in our study, the DBP did not, because the DBP is determined by several factors other than SVR—for example, the HR and the stiffness of the aorta.

This study demonstrated that a bolus injection of pimobendan decreases the LVEDP and the PCWP. The effect of a pimobendan injection on LVEDP is consistent with a previous study in anesthetized dogs, in which the LVEDP decreased with escalating doses of pimobendan (10, 20, 40 μg/kg/min) in a dose-dependent manner (18). In addition, the effect of a pimobendan injection on PCWP is consistent with a study in anesthetized dogs given escalating concentrations of pimobendan (10, 30, 100, and 300 μg/kg), in which the PCWP decreased as much as 84% from baseline at the highest dose (19). The ability of intravenous pimobendan to reduce both the LVEDP and the PCWP suggests its beneficial use in CHF, in which the LVEDP and the PCWP are elevated as a result of left ventricular failure resulting in pulmonary edema (28).

The single bolus of pimobendan (0.15 mg/kg) in this study caused the PQ interval to decrease below baseline levels, whereas the HR increased. The PQ duration, or the time that an impulse uses for conduction in the intra-atrium and the delay within the atrioventricular node, is HR dependent (29). Thus, this abbreviation of PQ interval can be partly explained by the relationship between the HR and the PQ interval. Our findings on the duration of the PQ interval are consistent with previous published data in dogs (30, 31). A previous study in beagle dogs instrumented with radiotelemetry showed that the PQ interval demonstrates an almost linear inverse relationship with HR (31). In addition, the shortening of the PQ interval may be attributed to activation of the L-type calcium current by pimobendan. In a study of rat ventricular myocytes, PDE-3 and phosphodiesterase IV inhibitors were identified as the dominant phosphodiesterase subtypes that enhance the L-type calcium current (32), suggesting that an acute effect of pimobendan at a dose of 0.15 mg/kg may significantly alter calcium channels. The elevating HR can result from an increase in cAMP, the intracellular secondary messenger, as a result of PDE-3 inhibition. The cAMP increases could activate funny currents and L-type calcium currents (33). As a result, the action potential of the pacemaker cells could be generated more frequently (34). An increase of HR in this study was consistent with previous studies in anesthetized dogs (18, 19). Although the elevating HR can exacerbate heart failure the beneficial effects of pimobendan on positive inotropism outweigh the disadvantage, which supports the use of pimobendan in acute congestive heart failure.

Oral pimobendan has been recommended for management of CHF in dogs for more than a decade (6, 8), and the PK of this drug have been investigated in several species, including humans, pigs, dogs, and cats. However, because the intravenous, injectable form of pimobendan is relatively new, the PK data of this preparation remains limited. To our knowledge, the PK profiles of pimobendan at its manufacturer-recommended dose (0.15 mg/kg intravenously) has limited information. The Vd of pimobendan in this study is 8.9 L/kg. The Vd of pimobendan documented in the package insert was 2.6 L/kg. This variance could be due to the study design, the signalment of the dogs, or the samples in each experiment. Our study was performed in dogs under anesthesia for at least 2 h, which may have affected the PK properties of the drug and its metabolite. According to the package insert, the plasma elimination half-life of pimobendan is 0.4 ± 0.1 h, the clearance is 90 ± 19 mL/min/kg, and the MRT is short, at 0.5 ± 0.1 h. Our study reported the clearance of pimobendan as 5.8 ± 2.3 L/kg/h, which is relatively similar to that of package insert, but the half-life of pimobendan observed in our study is quite different from that of the package insert. Pimobendan is a known substrate for cytochrome P450 1A2; therefore, the non-steroidal anti-inflammatory drug used during the surgical procedure in this study may have altered the elimination duration and other PK parameters of pimobendan (35). Furthermore, a previous publication suggests that generalized anesthesia may prolong the time course of PK parameters (36).

In this study, the injectable pimobendan provides immediately positive inotropic effect which is suitable for dogs presenting with acute CHF. Previous study in healthy dogs demonstrated that the rectal administration of pimobendan at a dose of 0.5 mg/kg provides rapid absorption and achieves therapeutic plasma concentration which may be suitable for dog with CHF (37). In that study, the T_max_ and C_max_ of ODMP were 1.7 ± 1.1 h and 8.8 ± 4.8 ng/mL, respectively. In the current study, pimobendan was given by injection; therefore, the C_max_ of ODMP is 3.4 times higher while the T_max_ is 5.6 times faster than those of the previous study. In addition, the half-life of pimobendan and ODMP in this study was shorter while the AUC was presumably the same level based on data provided in the previous study (37).

This study has some limitations; therefore, the results must be interpreted with caution. First, the dogs were anesthetized and catheterized to observe the cardiac function and hemodynamic changes during the first 2 h of a PK-PD study. The slightly hypotensive status was observed at the beginning of the study which may be due to isoflurane-induced vasodilation (38). This small hypotension may affect the degree of responses of BP and other variables to intravenous pimobendan; however, it does not impact the conclusion of the current study. Furthermore, the PK parameters may have been affected by those procedures. Nevertheless, dogs were anesthetized with isoflurane inhalation. This anesthetic agent is mainly distributed into the brain with minimal level in blood (39–41). In addition, there is minimal reports of isoflurane on the interference of protein binding or pimobendan clearance. Second, the planned control group of anesthetized dogs receiving the vehicle did not take place in this study at the recommendation of the Institutional Animal Care and Use Committee of Chulalongkorn University, which was concerned about the 3R concept (i.e., replacement, reduction, and refinement). In our previously published study that used anesthetized dogs with the same setup, we saw no substantial change among parameters of ECG, hemodynamics, and cardiac functions for at least 150 min after the stabilization period in vehicle-treated dogs (15). Last, this study used healthy dogs; the effect of intravenous pimobendan and its metabolite may differ during clinical scenarios.

In conclusion, this study demonstrated significant acute cardiovascular effects of a bolus pimobendan in healthy animals. In response to intravenous pimobendan at the recommended dose for dogs, cardiac contraction increased and cardiac relaxation developed quickly after injection. The CO increased, but both SVR and PVR decreased. Blood pressure levels increased gradually, whereas the LVEDP, PCWP, RAP, and PAP decreased gradually. In addition, no incidence of arrhythmia had been observed. These mechanisms support the use of injectable pimobendan to treat acute CHF. Additional studies are warranted to describe the PK and PD of injectable pimobendan in dogs with heart failure.

## Data Availability Statement

The original contributions presented in the study are included in the article/supplementary material, further inquiries can be directed to the corresponding author/s.

## Ethics Statement

The animal study was reviewed and approved by The Institutional Animal Care and Use Committee, Chulalongkorn University Laboratory Animal Center.

## Author Contributions

PP contributed to performing experiments, analyzing the data, interpreting the data, and drafting the manuscript. LT, PB, and NS contributed to performing experiments and collecting data. TB and PK contributed to performing PK analysis and drafting the manuscript. AK contributed to the study design, to data analysis, and to drafting and revising the manuscript. All authors contributed to the article and approved the submitted version.

## Conflict of Interest

The authors declare that the research was conducted in the absence of any commercial or financial relationships that could be construed as a potential conflict of interest.

## Publisher's Note

All claims expressed in this article are solely those of the authors and do not necessarily represent those of their affiliated organizations, or those of the publisher, the editors and the reviewers. Any product that may be evaluated in this article, or claim that may be made by its manufacturer, is not guaranteed or endorsed by the publisher.
